# Preparation of Flexible Substrate Electrode for Supercapacitor With High-Performance MnO_2_ Stalagmite Nanorod Arrays

**DOI:** 10.3389/fchem.2019.00338

**Published:** 2019-05-14

**Authors:** Yuanyu Ge, Xianfeng Wang, Tao Zhao

**Affiliations:** College of Chemistry, Chemical Engineering and Biotechnology, Donghua University, Shanghai, China

**Keywords:** large-area, manganese dioxide, stalagmite nanorod arrays, supercapacitors, flexible electrode

## Abstract

A large-area MnO_2_ stalagmite nanorod arrays (SNAs) growing vertically on flexible substrates were successfully fabricated by an easy heat-electrodeposition method. The large specific capacitance (646.4 F g^−1^ at 500 mA g^−1^) and excellent rate capability (42.3% retention with 40 times of increase) indicate that the prepared MnO_2_ SNAs flexible electrode has outstanding electrochemical performance. Furthermore, after 5,000 repetitions of CV tests, the overall specific capacitance could retain ~101.2% compared with the initial value meant a long cycling life. These outstanding properties could be ascribed to the effective conductive transport path between Ni substrate and MnO_2_ nanorods, and owing to the stalagmite like structure of MnO_2_ nanorods, the exposed sufficient active sites are beneficial to the electrolyte infiltration.

## Introduction

The expanding requirement for energy consumption has stimulated the development of electrochemical energy storage devices (Simon and Gogotsi, 2008). Supercapacitors (SCs) occupy an important position in the field of energy storage devices due to their high power density, high-speed of charging and discharging and long cycle life (Burke, 2000). Nowadays, the development of supercapacitors to higher mechanical flexibility has become one of the important trends. It is shown that materials, such as graphene, transition metal dichalcogenides, MXenes (Han et al., 2018), and nanocellulose-graphene composites (Xing et al., 2019) could be used to construct nanomaterials-based flexible electrodes with high performance for supercapacitors, and there were relationships between structures and properties.

There are many different SCs electrode materials, such as carbon-based material (Li G. et al., 2018; Takeuchi et al., 2018) for the electric double layer capacitors (EDLCs), transition metal oxides, nitrides (Yi et al., 2018) and nickel based materials (Feng et al., 2014; Li et al., 2018) for the pseudocapacitors (PCs). Manganese dioxide (MnO_2_) is one of the rapid developed metal oxide electrode materials in recent years (Wang et al., 2015). α-MnO_2_ has a high specific capacitance among the various crystallographic structures of MnO_2_, which is mainly due to its largest tunnel (Sanger et al., 2016) that can store more foreign cations for charge balance. More importantly, the electrolytes used for MnO_2_ electrodes are non-corrosive neutral solutions, which is green and fit for environmental protection (Li et al., 2012).

As we know, the effects of active materials on the performance of SCs include morphology, structure, contact with collector plate and active sites, etc. Unfortunately, during the redox reaction, the electrons transport of MnO_2_ electrodes often restricted by its high electrical resistivity. In addition, the defect of the conventional powder electrode preparation is that the generated 'dead volume' by the bonding process of the active material to substrate could make a deterioration in the electrochemical properties of the electrodes (Liu et al., 2016). To solve the defects noted above, it could be a feasible way to manufacture binder-free MnO_2_ supporting on 3D conductive substrates. The benefits of the 3D binder-free electrode are the enhanced electron transport and sufficient free space between the nanostructures, which contribute to the generation of more active sites for Faradaic reaction. Li et al. prepared the Ni foam-based ultrafine MnO_2_ nanobelts with capacitance of 509 F g^−1^ at 0.2 A g^−1^ (Li et al., 2013). Davoglio et al. (2018) synthetized the α-MnO_2_ particles at the aqueous-organic interface with capacitance of 289 F g^−1^ at 0.5 A g^−1^. Although some achievements have been made in the preparation of MnO_2_ electrodes on conductive substrates, there is still great potential to develop easy methods for the preparation of high performance MnO_2_ nanowires with various forms.

In this paper, the heat-electrodeposition method was adopted to formulate a nanoarrays of stalagmite like MnO_2_ on the flexible substrates. The prepared electrode gets capacitance of 646.4 F g^−1^ (500 mA g^−1^) and 42.3% retention (current density increased 40 times) for a remarkable rate capability. And the total capacitance retention rate after 5,000 cycles is ~101.2%. Furthermore, to verify the generality of the synthesis method, another flexible activated carbon fiber (ACF) was also used as the substrate for the growth of MnO_2_ nanoarrays.

## Experimental

### Material Preparation

The preparing method in detail was: the heat-electrodeposition process carried on a 3D porous Ni foam. Before electrodeposition, the Ni foam was cut into ~ 3 × 1 cm^2^, and then immersed into a 5 mol/L HCl solutions along with supersonic wave treatment for 10 min to dissolve the NiO layer on the surface. The Ni foam obtained from the previous step was rinsed to neutral with distilled water, and then subjected to vacuum drying (60°C, 4 h). The heat-electrodeposition occurred in a cell with the water bath. The composition of the electrolyte was as follows: Mn(CH_3_COO)_2_ (0.01 M), CH_3_COONH_4_ (0.02 M) and dimethylsulfoxide (DMSO, 10 vol.%). The corresponding working electrode, the counter electrode and the reference electrode were the treated Ni foam, the Pt plate (1.5 × 1.5 cm^2^) and saturated calomel electrode (SCE), respectively. The heat-electrodeposition condition was applied at a constant current (0.5 mA cm^−2^) by the Autolab electrochemical workstation at ~ 80°C for 60 min. After that rinsed the obtained sample to neutral and placed it in a 60°C vacuum dryer for 4 h. Finally, the sample was calcined in N_2_ atmosphere (heating-up 0.5°C min^−1^, 250°C, 2 h). The weight gain of the sample after the deposition was the active matter weight.

### Material Characterizations

In order to analyze the samples qualitatively, the X-ray diffractometer (XRD; Rigaku D/max-2550 PC, Cu-Kα radiation) spectrum was utilized. To observe the microstructures, scanning electron microscope (SEM, Hitachi S-4800) and transmission electron microscope (TEM, JEOL JEM-2100F) were adopted. Mass weighing (Mettler Toledo XS105DU, δ = 0.01 mg).

### Electrochemical Measurements

The Electrochemical Workstation (Autolab PGSTAT302N, electrolyte 0.5 M Na_2_SO_4_) was used to measure electrochemical performance. In the test, the obtained MnO_2_ electrode (~1 cm^2^) was used as the working electrode. The counter and reference electrode were the same as mentioned above. Specific capacitance calculation (Guan et al., 2017):

(1) $$\begin{array}{r} C=I\cdot \Delta t/\left(\Delta V\cdot m\right) \end{array}$$

where *C* (F g^−1^) is the specific capacitance, *I* (A) is the discharge current, Δ*t* (s) is the discharge time consumed in the potential range of Δ*V*, Δ*V* (V) is the potential window, *m* (g) is the mass of the active materials.

The weight of the 1 cm^2^ MnO_2_ electrode was ~ 1.32 mg. The cyclic voltammetry (CV) potential window was −0.1 to 0.9 V. The scan rates increased from 1 to 100 mV s^−1^. The galvanostatic charge-discharge (GCD) curves were measured under current densities from 0.5 to 20 A g^−1^. The cycle life was obtained by CV test (50 mV s^−1^) with repetitions of 5,000.

## Results and Discussions

**Figure 1:**
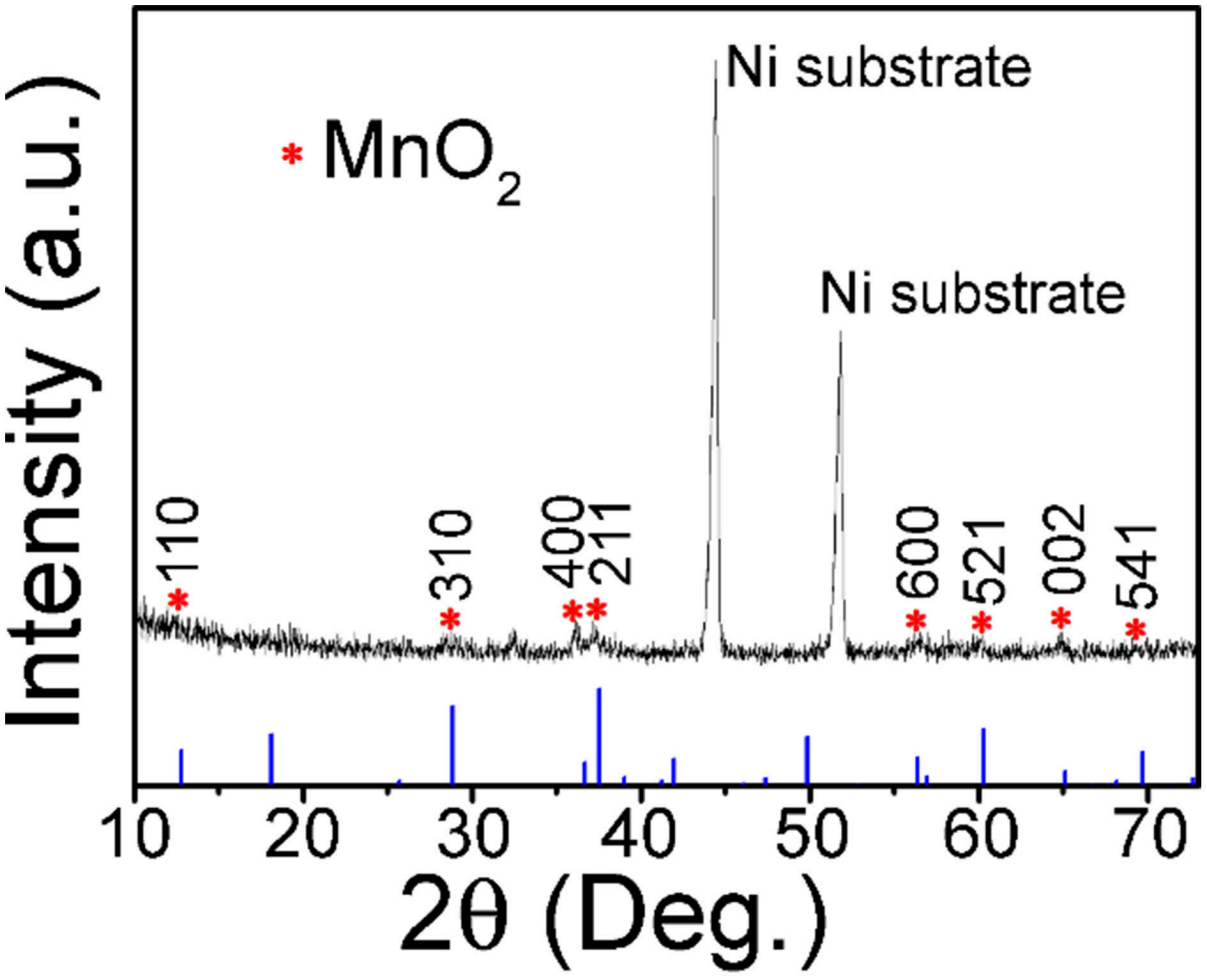
XRD pattern of the prepared MnO_2_ sample.

Except for the two strong peaks of 3D Ni foam substrate, the XRD diffraction pattern in Figure 1 shows that other peaks at 12.8, 28.8, 36.7, 37.5, 56.4, 60.3, 65.1, and 69.7°, which are characteristic (110), (310), (400), (211), (600), (521), (002), and (541) reflections of α-MnO_2_ (JCPDS 44-0141, a = b = 9.785 Å and c = 2.863 Å), respectively.

**Figure 2:**
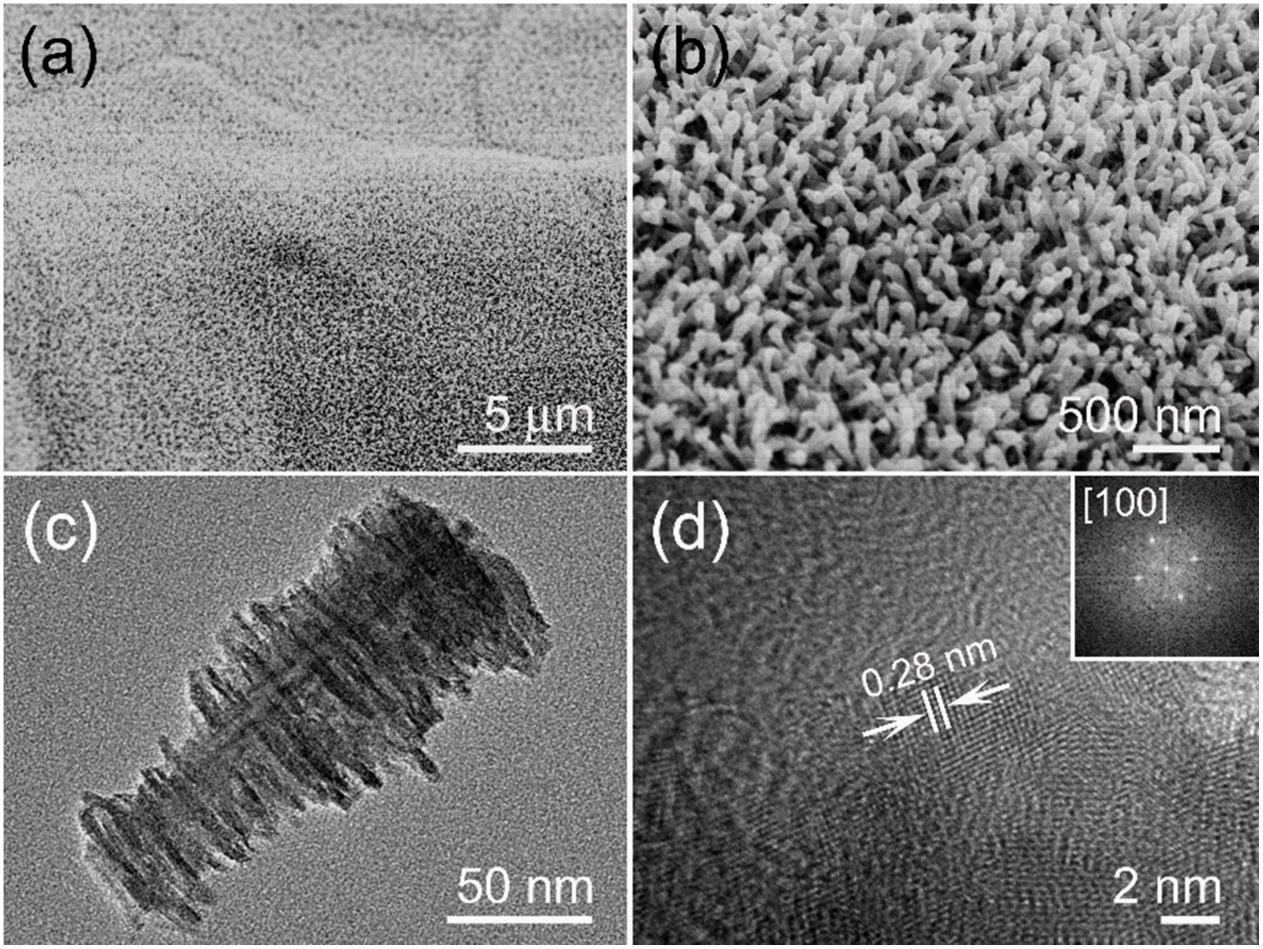
**(a)** Low and **(b)** enlarged SEM photos of MnO_2_ nanoarrays. **(c)** TEM image of the stalagmite MnO_2_ nanorod. **(d)** Lattice resolved HRTEM image of a MnO_2_ nanorod, an upper right inset showing its corresponding FFT pattern.

An aligned and dense MnO_2_ nanoarray is presented in Figure 2a and there are no macroscopic defects among them. The enlarged SEM image of Figure 2b shows a highly open structure was formed by a vertical MnO_2_ arrays on the substrate, which is conducive to the full entry of electrolytes. The TEM characterization was performed in order to get more structural information of the MnO_2_ arrays. Figure 2c is a single stalagmite like MnO_2_ nanorod which appears to be truncated cone-shaped with burrs on its surface. The typical diameters are ~30 nm of the root and ~80 nm of the top, and the length is up to ~180 nm. HRTEM image of the single MnO_2_ nanorod edge (Figure 2d) suggests that the surface is clearly with uniform single crystal and the interplanar spacing of 0.28 nm matches the (001) lattice plane of α-MnO_2_ crystal. In addition, FFT diffraction pattern can also be associated with [100] zone axis of α-MnO_2_ crystal (inset of Figure 2d).

**Figure 3:**
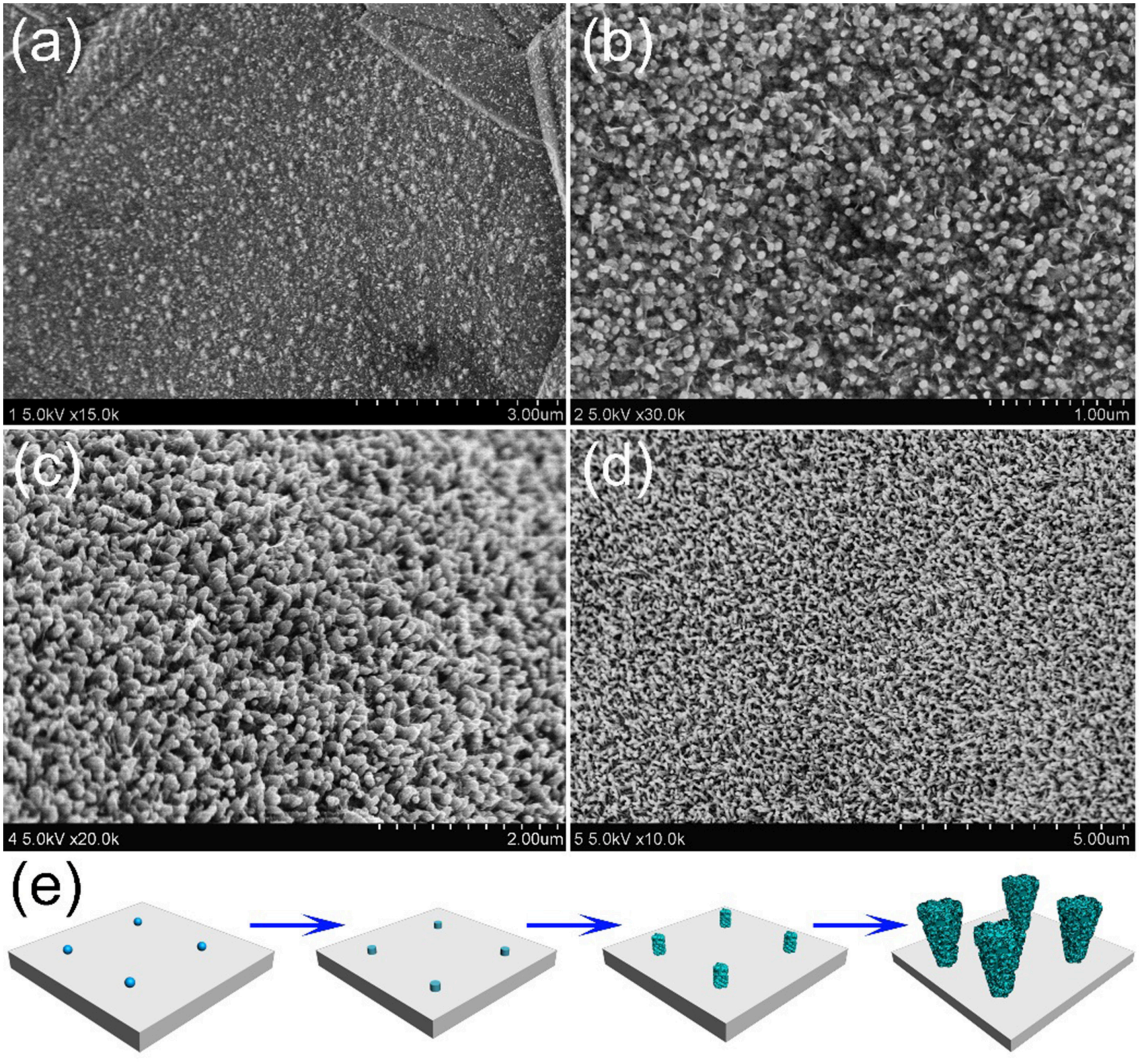
SEM images of the stalagmite MnO_2_ nanorod arrays for different electrodepositing time: **(a)** 2 min, **(b)** 20 min, **(c)** 40 min, and **(d)** 60 min; **(e)** Schematic diagram of the MnO_2_ SNAs formation.

In order to understand how the unique structure of stalagmite MnO_2_ nanorod arrays (MnO_2_ SNAs) was formed, the time-dependent electrodepositing experiments were carried out through controlling the electrodeposit reaction time. Figure 3a shows that after deposition of 2 min, nanoparticles can be seen on the surface of the substrate with the diameters of 10–20 nm. When the reaction time increases to 20 min, lots of well-distributed cylindrical nanorods began to appear, as displayed in Figure 3b. After doubling the reaction time, some irregular burrs were observed around the increased nanorods (Figure 3c). As shown in Figure 3d, after 60 min of the electrodeposition, it is found that large-scale and uniform nanorod arrays were formed. The change of the morphology of the samples after different electrochemical deposition time could help us estimate the formation process of the MnO_2_ nanorod arrays. In the early stage of the reaction, the formation of the nucleus in the precursor solution and the 1D growth behavior of Mn^2+^ (Ding et al., 2011) occurs successively, which result in the deposition of some tiny nanorods randomly and fast all over the surface. As the deposition intensifies, nanoparticles develop into nanorods with burrs, similar to stalagmite. When the reaction time reached to 1 h, the nanorods presented a truncated cone-shaped and formed a high-density array. The formation process of MnO_2_ SNAs was elucidated in Figure 3e.

Furthermore, to verify the generality of the synthesis method, another flexible ACF substrate was chosen to replace the Ni foam. Figure S1 shows that the dense needle-like MnO_2_ nanorod arrays were grown on the ACF, which proves that the heat-electrodeposition method could be used to fabricate electrodes with different flexible substrates.

**Figure 4:**
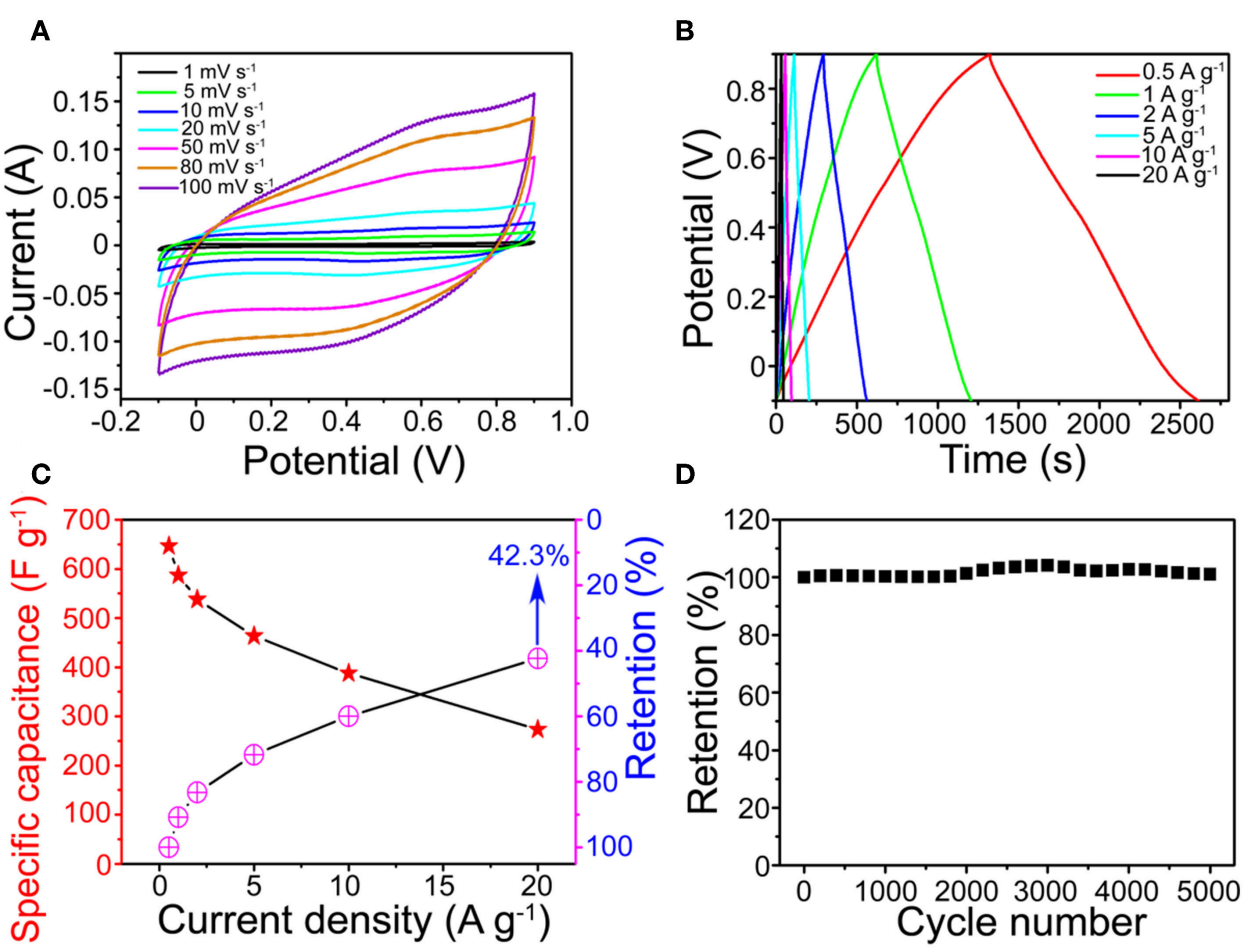
**(A)** CV and **(B)** GCD curves of the MnO_2_ SNAs electrode. **(C)** Specific capacitance and retention of the MnO_2_ SNAs electrode. **(D)** CV cycling performance.

Figure 4A shows that the MnO_2_ SNAs electrode CV with scanning rate from 1 to 100 mV s^−1^ were approximate to rectangle and symmetrical. All CV curves feature exhibits the pseudocapacitive nature (Figure S3) and a high-speed charge and discharge process (Hu et al., 2016). The possible reason for this phenomenon could be that the active sites in the microstructures of the stalagmite MnO_2_ SNAs were fully in contact with electrolytes. The symmetrical GCD curves of the MnO_2_ SNAs shown in Figure 4B revealed that the reversible redox reaction and good electrochemical capacitance characteristics of the system. In addition, by comparing the specific capacitance of the Ni foam substrate and the prepared MnO_2_ SNAs electrode in Figure S2, it is found that the substrate has little effect on the capacitance value.

In Figure 4C, the calculated specific capacitance of the electrode were 646.4, 587.1, 538.1, 463.6, 387.6, and 273.6 F g^−1^ (Red Star), respectively, which were higher than reported works with similar MnO_2_ structure (Li et al., 2013; Wang et al., 2017; Davoglio et al., 2018). Figure 4C also shows that the capacitance retention increased with the increase of current density. When it increased 40 times from 0.5 to 20 A g^−1^, the corresponding specific capacitance reduced from 646.4 to 273.6 F g^−1^ with the retention of 42.3%. The excellent rate capability indicates the potential of the MnO_2_ SNAs electrode in high-power applications, and it could be caused by the unique open structure. The advantages of the open structure are that it is favorable for electrolyte infiltration and it owns large electrolytic accessible area, which not only promote redox reaction but also facilitates intercalation and de-intercalation of active species.

Figure 4D shows that a beneficial cycle life of the electrode and the capacitances retention enlarged during the initial 3,000 loops. The possible explanation is that the initially inactive part of the MnO_2_ SNAs electrode was activated by the permeated electrolyte and then the specific capacitance is increased (Xia et al., 2012).

After 5,000 cycles, a high specific capacitance retention of 101.2% indicated that the cyclical stability of the MnO_2_ SNAs electrode was good. The strong contact between the active matters and the substrate facilitating the collection and enhancement of the electron participation reaction could be an important reason for the effective cycle stability. Thus, it is concluded that the electrode material of the as-synthesized MnO_2_ SNAs demonstrates an excellent cycle life.

## Conclusion

In conclusion, the stalagmite MnO_2_ nanorod arrays successfully grew on the flexible substrate by heat-electrochemical deposition method. The prepared MnO_2_ SNAs electrode has the high specific capacitance, the outstanding rate capability and the long cycle life, all of which all suggest its excellent electrochemical performance. In addition, this approach could pave the way for a facile low-temperature heat synthetic route for generating a variety of metal oxides arrays flexible substrate electrode.

## Data Availability

The raw data supporting the conclusions of this manuscript will be made available by the authors, without undue reservation, to any qualified researcher.

## Author Contributions

YG did the experiments and described the images of figures. XW helping with writing. TZ was the supervisor of this research work. All authors participated in the analysis of experimental data and manuscript preparation.

### Conflict of Interest Statement

The authors declare that the research was conducted in the absence of any commercial or financial relationships that could be construed as a potential conflict of interest.
