# Supplementary material for: Preparation of Flexible Substrate Electrode for Supercapacitor With High-Performance MnO2 Stalagmite Nanorod Arrays
Source: Front Chem. 2019 May 14;7:338. doi: 10.3389/fchem.2019.00338 (PMC6527770; doi:10.3389/fchem.2019.00338)
Supplement: Supplementary file 1 [file Table_1.DOC]

Supplementary Material

# Preparation of Flexible Substrate Electrode for Supercapacitor with High-performance MnO_2_ Stalagmite Nanorod Arrays

Yuanyu Ge, Xianfeng Wang and Tao Zhao*

College of Chemistry, Chemical Engineering and Biotechnology, Donghua University, Shanghai 201620, China.

* Corresponding authors.

E-mail addresses: tzhao@dhu.edu.cn


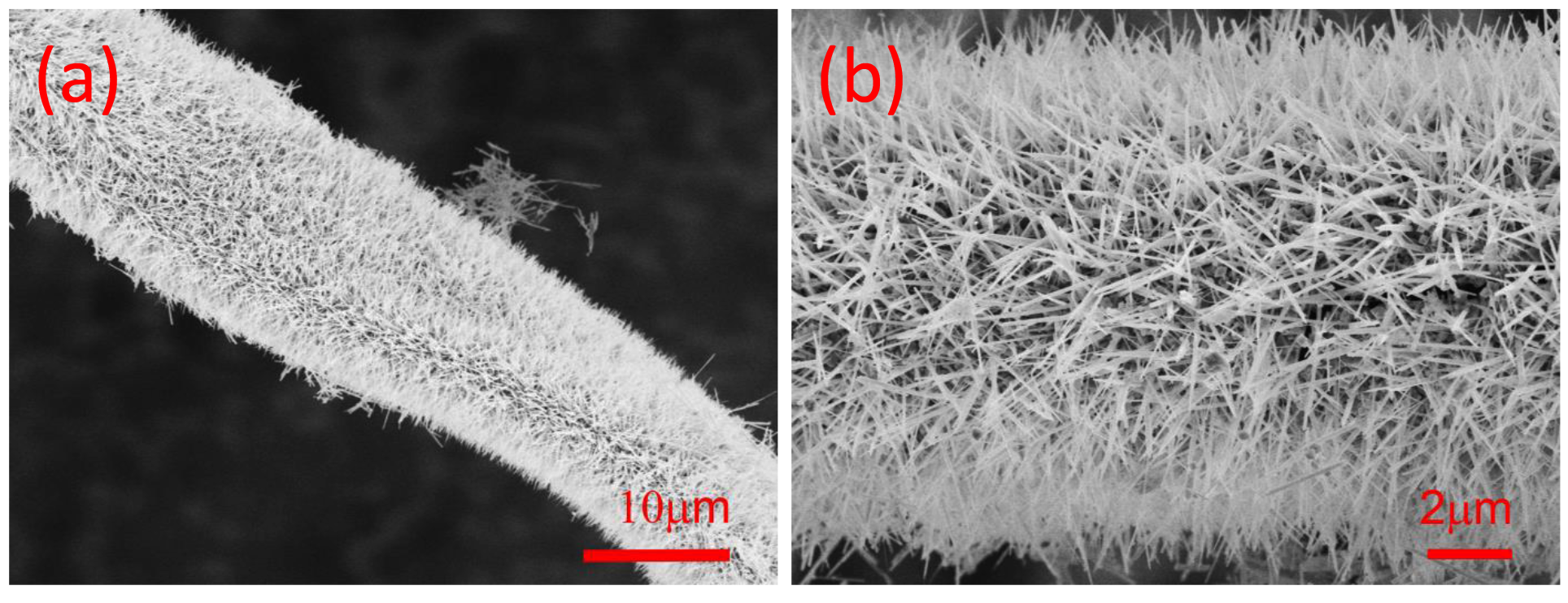


**Fig. S1** (a) Low and (b) High magnification SEM image of the MnO_2_ nanorod arrays on the ACF substrate, the ACF surface was evenly covered by the MnO_2_ nanorod arrays with a dense needle-like structure.


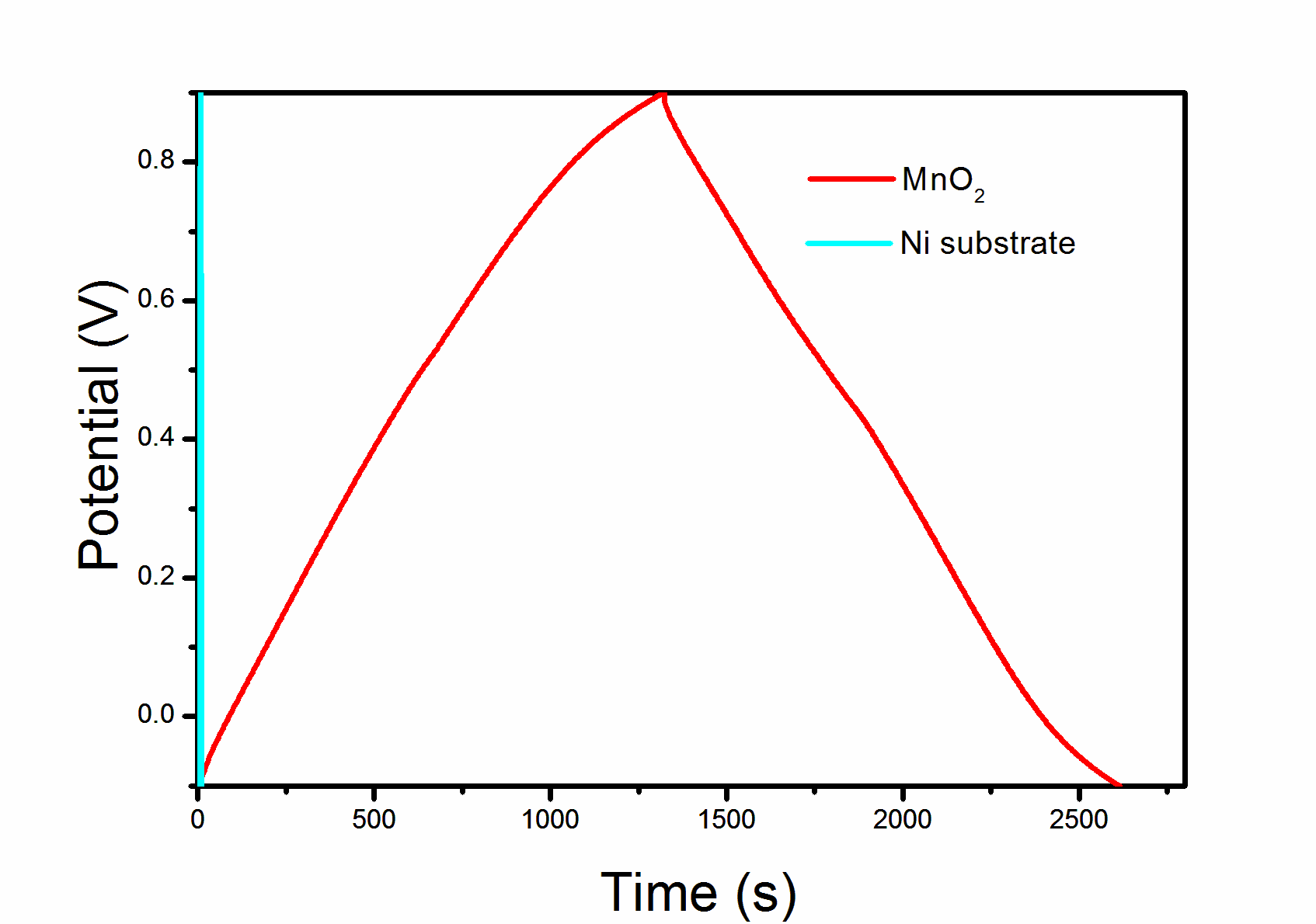


**Fig. S2** Galvanostatic charge-discharge curves of the MnO_2_ SNAs and the bare Ni substrate at a current density of 500 mA g^-1^.





**Fig. S3** Fitting curve of scanning speed (v) and current (i)
